# Temperature-dependent expression of different guanine-plus-cytosine content 16S rRNA genes in *Haloarcula* strains of the class *Halobacteria*

**DOI:** 10.1007/s10482-018-1144-3

**Published:** 2018-08-20

**Authors:** Yu Sato, Hiroyuki Kimura

**Affiliations:** 1 Department of Environment and Energy Systems, Graduate School of Science and Technology, Shizuoka University, Shizuoka, 422-8529 Japan; 2 Department of Geosciences, Faculty of Science, Shizuoka University, 836 Oya, Suruga-ku, Shizuoka, 422-8529 Japan; 3 Research Institute of Green Science and Technology, Shizuoka University, Shizuoka, 422-8529 Japan

**Keywords:** 16S rRNA gene, Expression, *Haloarcula*, Guanine-plus-cytosine content, Thermal stability, Temperature

## Abstract

*Haloarcula* strains, which are halophilic archaea, harbour two to three copies of 16S rRNA genes (*rrsA*, *rrsB* and *rrsC*) in their genomes. While *rrsB* and *rrsC* (*rrsBC*) show almost identical sequences, *rrsA* shows 4–6% sequence difference and 1–3% guanine-plus-cytosine content (*P*_*GC*_) difference compared to *rrsBC*. Based on the strong correlation between the *P*_*GC*_ of 16S rRNA genes and the growth temperatures of the prokaryotes, we hypothesised that high-*P*_*GC*_*rrsA* and low-*P*_*GC*_*rrsBC* are expressed at high and low temperatures, respectively. To verify the hypothesis, we performed sequence analyses and expression surveys of each 16S rRNA gene in eight *Haloarcula* strains. The secondary structure prediction of the 16S rRNA via computer simulation showed that the structural stability of 16S rRNAs transcribed from *rrsA* was higher than that of 16S rRNAs transcribed from *rrsBC*. We measured expression levels of *rrsA* and *rrsBC* under various temperature conditions by reverse-transcriptase quantitative PCR. The expression ratio of high-*P*_*GC*_*rrsA* to low-*P*_*GC*_*rrsBC* increased with cultivation temperatures in seven of eight *Haloarcula* strains. Our results suggest that the transcription of high-*P*_*GC*_*rrsA* and low-*P*_*GC*_*rrsBC* may be regulated in response to environmental temperature, and that 16S rRNAs transcribed from high-*P*_*GC*_*rrsA* function under high temperature conditions close to the maximum growth temperature.

**Electronic supplementary material:**

The online version of this article (10.1007/s10482-018-1144-3) contains supplementary material, which is available to authorized users.

## Introduction

Prokaryotes harbour 16S rRNA genes encoding rRNA in the small subunit of the prokaryotic ribosome, and the sequences are highly conserved. Therefore, the 16S rRNA gene sequence is generally used for the identification of prokaryotes and for the assessment of prokaryotic diversity in various environments (Narihiro and Kamagata 2017; Yabe et al. 2017). A high percentage of prokaryotic genomes (83%) contain two or more copies of 16S rRNA genes, and the sequences are nearly identical among the copies of 16S rRNA genes (Sun et al. 2013). However, recent genome analyses revealed that some prokaryotes show intragenomic heterogeneity of 16S rRNA genes (Větrovský and Baldrian 2013; Sun et al. 2013). In particular, halophilic archaea of the genus *Haloarcula* have long been known to harbour significantly different sequences of 16S rRNA genes in their genomes (Mevarech et al. 1989; Acinas et al. 2004; Boucher et al. 2004; Pei et al. 2010; Sun et al. 2013).

*Haloarcula* strains have been isolated from solar salterns and salt lakes in deserts (Juez et al. 1986; Oren et al. 1990), where large day-night temperature variations are observed (Wieland et al. 2005; Sima et al. 2013; Andrade et al. 2015; Naghoni et al. 2017). These strains have two or three copies of rRNA operons (*rrnA*, *rrnB* and *rrnC*) in their genomes (Baliga et al. 2004; Liu et al. 2011; Ding et al. 2014; Yun et al. 2015; Sato et al. 2017). Each rRNA operon contains a 5S rRNA gene, a 16S rRNA gene (*rrsA*, *rrsB* or *rrsC*, respectively) and a 23S rRNA gene. The 5S rRNA gene sequences are almost identical among the rRNA operons. The 23S rRNA gene sequences show limited diversity (0.3–2%). For 16S rRNA gene sequences, *rrsB* and *rrsC* (*rrsBC*) show almost identical sequences, whereas *rrsA* shows different sequences (4–6%) from *rrsBC* (Mevarech et al. 1989; Mylvaganam and Dennis 1992; Dennis 1999; Sato et al. 2017). The different sequences of 16S rRNA genes were first reported in *Haloarcula marismortui* (Mevarech et al. 1989). Adjacent regions such as the putative promoter of 16S rRNA genes and the maturation process of the transcribed 16S rRNA have already been studied (Mylvaganam and Dennis 1992; Dennis et al. 1998; Dennis 1999; Amann et al. 2000). Previous studies showed that variation of the salinity in the medium did not change the expression level of each 16S rRNA gene in *Haloarcula* strains (López-López et al. 2007; Cui et al. 2009). However, it has been reported that variation of the cultivation temperature changed the expression level of each 16S rRNA gene in *H. marismortui* (López-López et al. 2007).

It has been shown that 16S rRNA gene sequences contain information regarding the thermal features of prokaryotes (Galtier and Lobry 1997; Khachane et al. 2005; Kimura et al. 2006; Wang et al. 2006; Kimura et al. 2010, 2013). Those findings were based on a high correlation between the guanine-plus-cytosine contents (*P*_*GC*_) of 16S rRNA genes and the growth temperatures of archaea; the *P*_*GC*_ of 16S rRNA genes in thermophilic and hyperthermophilic archaea tend to be high, whereas the *P*_*GC*_ of 16S rRNA genes in psychrophilic and mesophilic archaea tend to be comparatively low (Kimura et al. 2007). On the basis of the relationship between the *P*_*GC*_ of 16S rRNA gene sequences and the growth temperatures of archaea, Kimura et al. (2013) proposed the microbial molecular thermometer (MMT), which uses linear regression equations to infer the minimum (*T*_*min*_), optimal (*T*_*opt*_) and maximum (*T*_*max*_) growth temperatures of cultured and not-yet cultured archaea based on the *P*_*GC*_ value of each 16S rRNA gene.

Sato et al. (2017) used *Haloarcula hispanica* to verify a hypothesis that high-*P*_*GC*_*rrsA* (58.9%) and low-*P*_*GC*_*rrsBC* (56.4–56.5%) are expressed at high and low temperatures, respectively. They also investigated the physiological advantage of harbouring high- and low-*P*_*GC*_ 16S rRNA genes with respect to the growth of *H. hispanica* under various temperature conditions. An expression survey by reverse-transcriptase quantitative PCR (RT-qPCR) showed that the expression ratio of *rrsA* to *rrsBC* increased with the incubation temperature. At the *T*_*max*_, i.e., 50 °C, the expression level of *rrsA* was significantly higher than those of *rrsBC*. Sato et al. (2017) also constructed mutant strains with only one of three rRNA operons and cultivated the strains at various temperatures. The mutant strain harbouring only *rrnA* including high-*P*_*GC*_*rrsA* grew significantly slower than the wild-type strain at the *T*_*min*_ 25 °C, but it grew as fast as the wild-type at 45 °C (*T*_*opt*_) and 50 °C (*T*_*max*_). Sato et al. (2017) thus demonstrated that high-*P*_*GC*_ 16S rRNA is important for the rapid growth of *H. hispanica* at high temperature.

*Haloarcula* sp. CBA1115, *Haloarcula japonica*, *Haloarcula argentinensis*, *Haloarcula amylolytica*, *Haloarcula quadrata*, *Haloarcula vallismortis* and “*Haloarcula californiae*” also show different sequences of 16S rRNA genes as well as *H. marismortui* and *H. hispanica* (Javor et al. 1982; Takashina et al. 1990; Ihara et al. 1997; Oren et al. 1999; Yang et al. 2007; Yun et al. 2015). The sequence differences and the *P*_*GC*_ offsets between *rrsA* and *rrsB* or *rrsC* range from 4.8 to 5.6% and from 1.3 to 2.9%, respectively (Sato et al. 2017). However, the temperature-dependent expression of *rrsA* and *rrsBC* in the members of the genus *Haloarcula* are not yet well understood.

Here, we investigated eight *Haloarcula* strains harbouring different sequences of 16S rRNA genes in their genomes (Sato et al. 2017). We sequenced the 16S rRNA genes in each strain and estimated the *T*_*min*_, *T*_*opt*_ and *T*_*max*_ based on the *P*_*GC*_ of the 16S rRNA genes, using the MMT proposed by Kimura et al. (2013), in addition to measuring the growth temperatures by general cultivation methods. We also calculated the thermal stability of the secondary structure for each 16S rRNA transcribed from *rrsA*, *rrsB* or *rrsC* in the *Haloarcula* strains via computer simulation (in silico). The expression ratio of *rrsA* to *rrsBC* was determined under various temperature conditions by RT-qPCR. We used the MMT, secondary structure analysis and RT-qPCR to test our hypothesis that *Haloarcula* strains express and use high-*P*_*GC*_ 16S rRNAs at high temperature and low-*P*_*GC*_ 16S rRNAs at low temperature.

## Materials and methods

### *Haloarcula* strains and culture conditions

Eight *Haloarcula* strains were used in this study: *Haloarcula* sp. CBA1115 JCM 30477, *H*. *argentinensis* JCM 9737, *H. quadrata* JCM 11048, *H. vallismortis* JCM 8877, *H. amylolytica* JCM 13557, *H. japonica* JCM 7785, *H. hispanica* JCM 8911 and “*H. californiae*” JCM 8912 were obtained from the Japan Collection of Microorganisms (JCM; Ibaraki, Japan). Each strain was cultured using JCM Medium 307: 2 g of casamino acid (BD, Franklin Lakes, NJ, USA), 2 g of Bacto yeast extract (BD), 1 g of sodium glutamate, 3 g of trisodium citrate, 10 g of MgSO_4_·7H_2_O, 1 g of CaCl_2_·2H_2_O, 1 g of KCl, 200 g of NaCl, 0.36 mg of FeCl_2_·4H_2_O and 0.36 mg of MnCl_2_·4H_2_O per a litre of distilled water.

The *Haloarcula* strains were incubated in the dark in 60 ml screw-capped test tubes containing 20 ml of JCM Medium 307 with shaking at 120 or 180 rpm at 20, 25, 30, 35, 40, 45, 50, 55 and 60 °C. The culture experiment was performed in quadruplicate. The optical density at 660 nm (OD_660_) of the cultures was monitored using a Spectronic 200 spectrophotometer (Thermo Fisher Scientific, Waltham, MA, USA) against sterilised medium that was incubated under the same condition as a reference. The growth rates were calculated from OD_660_ values as described earlier (Sato et al. 2017). The actual *T*_*min*_, *T*_*opt*_ and *T*_*max*_ were determined based on the growth rate at each temperature.

### DNA extraction, 16S rRNA gene sequencing and sequence analyses

Cells of *Haloarcula* strains were inoculated into 60 ml screw-capped test tubes containing 20 ml of JCM Medium 307 and incubated in the dark with shaking at 120 or 180 rpm at 40 °C for 100–150 h. The cells were then collected by centrifugation at 12,000×*g* for 3 min, and the pelleted cells were stored at − 25 °C until DNA extraction.

DNA extraction from the pelleted cells was performed using the described methods with modifications (Tchinda et al. 2016; Sharmin et al. 2018). Briefly, the cells of *Haloarcula* strains were lysed by lysozyme and proteinase K. Bulk DNAs were extracted using both phenol/chloroform/isoamyl alcohol and chloroform/isoamyl alcohol and purified with ethanol precipitation. Next, 16S rRNA gene fragments were amplified by PCR with KOD -Plus- DNA polymerase (Toyobo, Osaka, Japan) from the bulk DNAs using a primer set 8aF/1448hR (Table 1). The primer 1448hR, which is specific for 16S rRNA genes in *Haloarcula* strains, was designed using Genetyx-Mac ver. 19.0.1 software (Genetyx, Tokyo, Japan).

**Table 1:** Primers used for PCR, sequencing and RT-qPCR in this study

Name	Sequence	Target (position)	References
8aF	5′-TCY GGT TGA TCC TGC C-3′	Archaeal 16S rRNA gene (3–18)	Lefèvre et al. (2011)
1448hR	5′-GGC TAC CTT GTT ACG ACT T-3′	16S rRNA gene of *Haloarcula* strains (1424–1442)	This study
109aF	5′-AMD GCT CAG TAA CAC GT-3′	Archaeal 16S rRNA gene (83–99)	Matsushita et al. (2016)
915aR	5′-GTG CTC CCC CGC CAA TTC CT-3′	Archaeal 16S rRNA gene (858–877)	Matsushita et al. (2018)
rrsAf	5′-CGT CCA GCG GAA ACT GTC CGG-3′	Partial sequence of *rrsA* in *Haloarcula* strains (569–589)	Sato et al. (2017)
rrsAr	5′-CCG TCG GGT CCG TCT TCC TGA G-3′	Partial sequence of *rrsA* in *Haloarcula* strains (674–695)	Sato et al. (2017)
rrsAf2	5′-CGT CCA GCG GAA ACT GTC AGG-3′	Partial sequence of *rrsA* in *Haloarcula quadrata* (569–589)	This study
rrsAr2	5′-CCG TCG GGT CCG TCT TCT TGA G-3′	Partial sequence of *rrsA* in *Haloarcula vallismortis* (674–695)	This study
rrsBCf	5′-GGC GTC CGG TGG AAA CTA CAC AG-3′	Partial sequence of *rrsBC* in *Haloarcula* strains (567–589)	Sato et al. (2017)
rrsBCr	5′-CAC TGT CGG GTC CGG TCT CTC AAC-3′	Partial sequence of *rrsBC* in *Haloarcula* strains (674–697)	Sato et al. (2017)

The PCR products were purified with a MicroSpin S-400 HR column (GE Healthcare, Little Chalfont, UK) or a QIAquick gel extraction kit (Qiagen, Hilden, Germany). The purified PCR products were cloned using a Zero Blunt TOPO PCR cloning kit (Life Technologies, Carlsbad, CA, USA) following the manufacturer’s protocol. Insert DNA in each clone was sequenced by the dideoxy cycle-sequencing method using an Applied Biosystems 3730*xl* DNA analyzer (Life Technologies). We checked the sequences with the Bellerophon program (Huber et al. 2004) and no chimeric products were detected. We determined the current nearest relative of each 16S rRNA gene by using the BLAST program (Altschul et al. 1990) and the sequences of *rrsA*, *rrsB* and *rrsC* in each strain were identified.

We deposited the 16S rRNA gene sequences of the *Haloarcula* strains in the DDBJ/EMBL/GenBank database under Accession Nos. LC085248, LC085249 and LC198781 to LC198794 (Table S1).

We also performed sequence analyses of 16S rRNA genes in other halophilic archaea. We obtained the sequences derived from 233 species in 58 genera in class *Halobacteria* from the DDBJ database and manually searched halophilic archaea with high intragenomic heterogeneity (more than 1%) of 16S rRNA genes. The sequence differences and *P*_*GC*_ offsets among copies of their 16S rRNA genes in the halophilic archaea were calculated using Genetyx-Mac ver. 19.0.1 (Genetyx).

### Estimation of growth temperatures

The 16S rRNA genes of thermophilic and hyperthermophilic archaea have higher *P*_*GC*_ values compared to those of psychrophilic and mesophilic archaea (Galtier and Lobry 1997; Khachane et al. 2005; Kimura et al. 2007). Based on the strong correlation between the *P*_*GC*_ values of 16S rRNA genes and the growth temperatures of archaea, linear regression equations to estimate the *T*_*min*_, *T*_*opt*_ and *T*_*max*_ of archaea based on *P*_*GC*_ of the 16S rRNA genes have been proposed (Kimura et al. 2013). We manually selected the internal sequences between the *Archaea*-specific primers 109aF and 915aR, used by Kimura et al. (2013), from the 16S rRNA gene sequences (Table 1). The *P*_*GC*_ values of the internal sequences were calculated using Genetyx-Mac ver. 19.0.1 (Genetyx). The *T*_*min*_, *T*_*opt*_ and *T*_*max*_ of eight *Haloarcula* strains were estimated based on the *P*_*GC*_ of *rrsA*, *rrsB* and *rrsC* using the linear regression equations as follows.

$$ \begin{aligned} T_{min} =\, & 4.38\left( { \pm \,0.14} \right)P_{GC} - 225.3\left( { \pm \,8.5} \right) \\ T_{opt} =\, & 4.98\left( { \pm \,0.10} \right)P_{GC} - 241.6\left( { \pm \,5.9} \right) \\ T_{max} =\, & 4.89\left( { \pm \,0.11} \right)P_{GC} - 228.2\left( { \pm \,6.6} \right) \\ \end{aligned} $$

### Prediction of 16S rRNA secondary structure and calculation for the structural stability

We used SSU-align ver. 0.1.1 to predict secondary structures of 16S rRNAs transcribed from *rrsA*, *rrsB* and *rrsC* based on the sequences determined in this study (Narwocki 2009). In addition, we calculated the minimal folding free energy (MFE, ∆*G* in kcal mol^−1^) value of each 16S rRNA with mfold web server ver. 2.3 (http://unafold.rna.albany.edu/?q=mfold/RNA-Folding-Form2.3) to investigate the thermal stability of each 16S rRNA secondary structure (Zuker 2003). The nearly full-length 16S rRNA sequences (approx. 1405 bp) determined in this study were used for the calculation. The sequence of *rrsC* in *H. hispanica* (Accession No. LC085247) contains Y (T or C) at position 392, and the nucleotide was C at the same position in *rrsC* obtained from the complete genome (Liu et al. 2011). We therefore calculated the MFE value of 16S rRNA transcribed from *rrsC* in *H. hispanica* by using the sequence modified from Y to C. The analyses using mfold web server were performed at the actual *T*_*min*_, *T*_*opt*_ and *T*_*max*_ of each strain. The significance of differences was determined by Student’s *t* test between MFE values of 16S rRNAs transcribed from *rrsA* and *rrsB* or *rrsC* under same temperature condition.

### Culture experiment to assess the expression of 16S rRNA genes

Cells of *Haloarcula* strains were inoculated into 60 ml screw-capped tubes containing 20 ml of JCM Medium 307. The cultures were incubated in the dark with shaking at 120 or 180 rpm at 20, 25, 30, 35, 40, 45, 50 and 55 °C. The OD_660_ of the cultures was monitored using a Spectronic 200 spectrophotometer (Thermo Fisher Scientific) against sterilised medium as a reference. When the cultures reached the early exponential growth phase (OD_660_ = 0.15–0.40), the cells were collected by centrifugation at 6270 or 12,000×*g* for 3 min. After the supernatants were discarded, the cells were treated with 100 µl of RNA later (Life Technologies) and stored at − 85 °C until RNA extraction.

### Total RNA extraction and cDNA synthesis

We extracted total RNA from the pelleted cells by using a *mir*Vana miRNA isolation kit (Ambion, Austin, TX, USA). Contaminating DNA in the RNA solution was removed with the TURBO DNA-free kit (Life Technologies). Total RNA was purified with an RNeasy MinElute cleanup kit (Qiagen). The quality and quantity of purified RNA were checked with both a NanoVue Plus spectrophotometer (GE Healthcare) and a 2100 Bioanalyzer (Agilent Technologies, Santa Clara, CA, USA). High-quality RNAs (RNA integrity number > 9.0) were used to synthesise the single-strand cDNA using a SuperScript III first strand synthesis system (Life Technologies). The cDNA was purified by a QIAquick PCR purification kit (Qiagen).

### Quantitative PCR (qPCR)

A specific primer set rrsAf/rrsAr was used to quantify the expression level of *rrsA* in *Haloarcula* sp. CBA1115, *H. argentinensis, H. amylolytica*, *H. japonica* and “*H. californiae*” (Table 1). To measure the expression level of *rrsA* in *H. quadrata* and *H. vallismortis*, we designed two primers, rrsAf2 and rrsAr2, based on the sequences of rrsAf and rrsAr, respectively (Table 1). The primer sets, i.e., rrsAf2/rrsAr and rrsAf/rrsAr2, were used to measure the expression level of *rrsA* in *H. quadrata* and *H. vallismortis*, respectively. The expression levels of *rrsBC* were measured with the specific primer set rrsBCf/rrsBCr (Table 1).

To check the selectivity of these primer sets, we performed a qPCR with diluted PCR products (1/100, 1/1000, 1/10,000 and 1/100,000) of *rrsA*, *rrsB* and *rrsC* (partial regions of 16S rRNA genes between the *Archaea*-specific primers, 109aF/915aR) amplified from the clone libraries constructed in this study. Each PCR mixture contained 2 µl of diluted PCR products (1/100, 1/1000, 1/10,000 or 1/100,000), 2 µl of each designed primer set (each 300 nM), 10 µl of PowerUP SYBR Green master mix (Life Technologies) and 4 µl of nuclease-free water. The qPCR was performed on an Applied Biosystems 7300 real time PCR system (Kataoka et al. 2018). The PCR cycling conditions included an initial step of 50 °C for 2 min and 95 °C for 2 min followed by 40 cycles of 95 °C for 15 s, 58 °C for 15 s and 72 °C for 1 min. Electrophoresis of PCR products was performed to check primer selectivity and no contamination.

The quantification of *rrsA* and *rrsBC* contained in the cDNA samples was performed by the qPCR method described above. Each cDNA sample was tenfold diluted. The standard curves were prepared from diluted PCR products (1/100, 1/1000, 1/10,000 and 1/100,000) of *rrsA*, *rrsB* and *rrsC* (partial regions of 16S rRNA genes between the *Archaea*-specific primers 109aF/915aR) as described above. Each PCR reaction was performed in triplicate for technical replicates. Quantification of 16S rRNAs in *H. japonica* and *H. argentinensis* was performed in two batches for biological replicates. The significance of differences was calculated by Student’s *t* test among the results under different temperature conditions.

## Results and discussion

### Actual growth temperatures of the *Haloarcula* strains

Maximum growth rate and OD_660_ value of *Haloarcula* strains at each temperature are shown in Figs. S1 and S2. The growth temperatures of the eight *Haloarcula* strains are summarised in Table 2. *H. quadrata* and “*H. californiae*” grew at 20–45 °C with optimal growth at 40 °C. This result is not consistent with an earlier study in which *H. quadrata* grew up to 55 °C (Oren et al. 1999). *H. argentinensis*, *H. vallismortis*, *H. amylolytica*, *H. japonica* and *H. hispanica* grew from 20 to 50 °C, with optimal growth at 45 °C. The growth temperatures of *Haloarcula* sp. CBA1115 ranged from 20 to 55 °C, and the *T*_*max*_ is the highest growth temperature among the eight *Haloarcula* strains examined here.

**Table 2:** Actual growth ranges and optima, *P*_*GC*_ of 16S rRNA genes (*rrs*) and estimated growth ranges and optima for *Haloarcula* strains

Strain	JCM no.	Actual growth ranges and optima^a^			16S rRNA		Estimated growth ranges and optima^d^		
		*T*_*min*_ (°C)	*T*_*opt*_ (°C)	*T*_*max*_ (°C)	Type	*P*_*GC*_^c^ (%)	*T*_*min*_ (°C)	*T*_*opt*_ (°C)	*T*_*max*_ (°C)
*Haloarcula amylolytica*	13557	20	45	50	*rrsA*	58.6	31.4 ± 16.7	50.3 ± 11.8	58.4 ± 13.1
					*rrsB*	56.2	21.0 ± 16.4	38.4 ± 11.5	46.7 ± 12.8
					*rrsC*	56.2	21.0 ± 16.4	38.4 ± 11.5	46.7 ± 12.8
*Haloarcula japonica*	7785	20	45	50	*rrsA*	58.5	30.9 ± 16.7	49.7 ± 11.7	57.9 ± 13.1
					*rrsC*	56.4	21.7 ± 16.4	39.3 ± 11.5	47.6 ± 12.8
*Haloarcula hispanica*	8911	20	45	50	*rrsA* ^b^	58.9	32.6 ± 16.7	51.6 ± 11.8	59.7 ± 13.1
					*rrsB* ^b^	56.5	22.2 ± 16.4	39.8 ± 11.6	48.1 ± 12.8
					*rrsC* ^b^	56.4	21.7 ± 16.4	39.3 ± 11.5	47.6 ± 12.8
*Haloarcula* sp. CBA1115	30477	20	50	55	*rrsA*	59.0	33.1 ± 16.7	52.2 ± 11.8	60.3 ± 13.1
					*rrsB*	56.1	20.4 ± 16.4	37.8 ± 11.5	46.1 ± 12.8
					*rrsC*	56.1	20.4 ± 16.4	37.8 ± 11.5	46.1 ± 12.8
*Haloarcula argentinensis*	9737	20	45	50	*rrsA*	58.5	30.9 ± 16.7	49.7 ± 11.7	57.8 ± 13.1
					*rrsB*	56.5	22.2 ± 16.4	39.8 ± 11.6	48.1 ± 12.8
*Haloarcula quadrata*	11048	20	40	45	*rrsA*	58.5	30.9 ± 16.7	49.7 ± 11.7	57.8 ± 13.1
					*rrsB*	56.7	23.2 ± 16.4	40.9 ± 11.6	49.2 ± 12.8
*Haloarcula vallismortis*	8877	20	45	50	*rrsA*	58.4	30.3 ± 16.7	49.1 ± 11.7	57.2 ± 13.0
					*rrsB*	57	24.3 ± 16.5	42.2 ± 11.6	50.4 ± 12.9
“*Haloarcula californiae*”	8912	20	40	45	*rrsA*	58.4	30.3 ± 16.7	49.1 ± 11.7	57.2 ± 13.0
					*rrsB*	56.4	21.5 ± 16.4	39.3 ± 11.5	47.6 ± 12.8

^a^Actual growth ranges and optima were determined by cultivation with JCM Medium 307 in this study

^b^16S rRNA gene sequences of *H. hispanica* were quoted from Sato et al. (2017)

^c^*P*_*GC*_ values were calculated from partial sequences of 16S rRNA genes between primers 109aF and 915aR

^d^Estimated growth temperatures were calculated from *P*_*GC*_ of *rrsA*, *rrsB* and *rrsC* using the microbial molecular thermometer (MMT) of Kimura et al. (2013)

### Sequence analyses of 16S rRNA genes in the *Haloarcula* strains and other halophilic archaea

Almost full-length 16S rRNA genes (1405 bp) were amplified from bulk DNAs of *Haloarcula* strains, and clone libraries were constructed. We randomly selected and sequenced a total of 16 clones in each clone library. The BLAST analysis revealed that the 16S rRNA genes mostly matched with *rrsA*, *rrsB* and *rrsC* of the *Haloarcula* strains registered in the DDBJ/EMBL/GenBank database (Table S1). While *rrsB* and *rrsC* show nearly identical sequences, the sequence of *rrsA* was 4.6–6.0% (65–85 bp) different from those of *rrsBC* in the eight *Haloarcula* strains.

We also performed sequence analyses of 16S rRNA genes in other halophilic archaea and found 26 strains (representing 14 genera) with highly different sequences of 16S rRNA genes (more than 1.0%) (Table S2). Notably, five strains belonging to genera *Halomicrobium* and *Halosimplex* (5.8–8.7%) show higher sequence differences of 16S rRNA genes compared with *Haloarcula* strains (4.8–5.6%). On the other hand, *P*_*GC*_ offsets among the 16S rRNA genes are less than 1.7% in these halophilic archaea compared with *Haloarcula* strains.

### Estimation of the growth temperatures based on the *P*_*GC*_ of the 16S rRNA genes

We selected the internal sequences between the *Archaea*-specific primers 109aF and 915aR (795 bp) to estimate the growth temperatures of the *Haloarcula* strains, using the linear regression equations proposed previously (Kimura et al. 2013). The *P*_*GC*_ of the internal sequences of *rrsA* ranged from 58.4 to 59.0% (Table 2). The *P*_*GC*_ of the internal sequences of *rrsB* and *rrsC* ranged from 56.1 to 57.0%. The minimum and maximum *P*_*GC*_ offsets between *rrsA* and *rrsB* were 1.4% and 2.9% in *H. vallismortis* and *Haloarcula* sp. CBA1115, respectively.

The approximate growth temperatures were estimated based on the *P*_*GC*_ values of the partial 16S rRNA gene sequences (Table 2). The *T*_*opt*_ estimated based on the *P*_*GC*_ of *rrsA* in each *Haloarcula* strain ranged from 49.1 to 52.2 °C, whereas the *T*_*opt*_ estimated based on the *P*_*GC*_ of *rrsB* or *rrsC* in each *Haloarcula* strain ranged from 37.8 to 42.2 °C. Actual *T*_*opt*_ were typically temperatures between the estimated *T*_*opt*_ from the *P*_*GC*_ of *rrsA* and *rrsBC*. The offsets between *T*_*opt*_ estimated based on *P*_*GC*_ of *rrsA* and *rrsBC* in *Haloarcula* strains were 6.9–14.4 °C. These results indicate that harbouring high- and low-*P*_*GC*_ 16S rRNA genes may enable *Haloarcula* strains to rapidly grow under a wide range of temperature conditions.

### Prediction of the secondary structures of 16S rRNAs in the *Haloarcula* strains

**Fig. 1:**
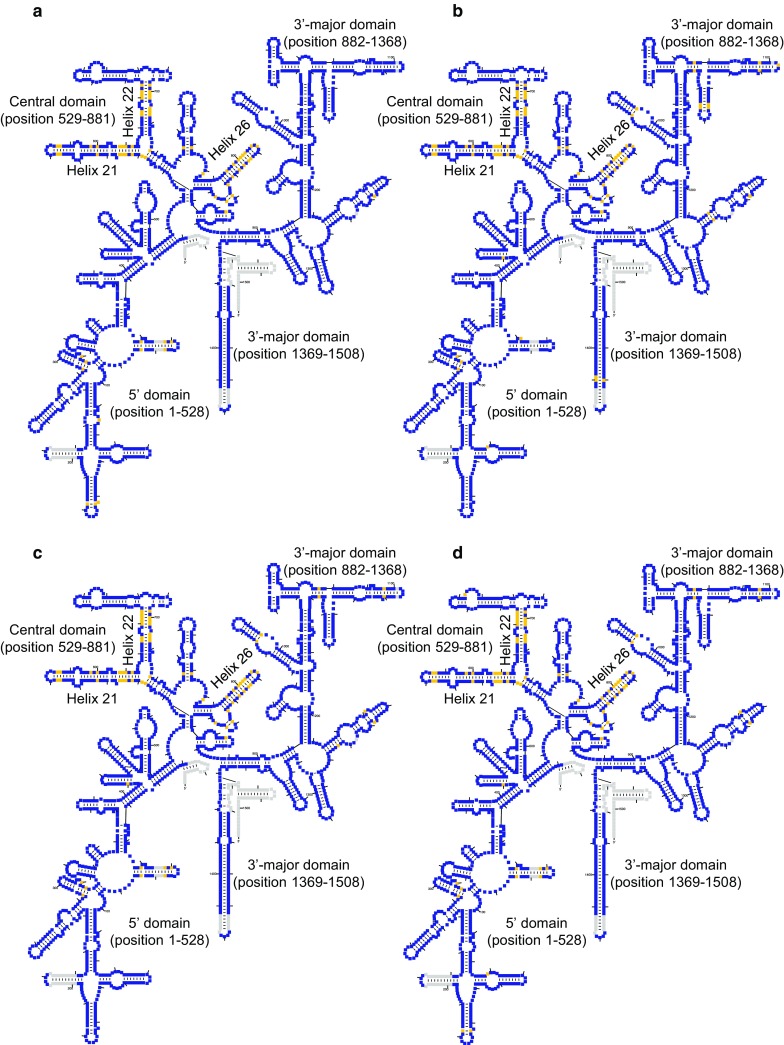
Secondary structure of 16S rRNAs in **a***Haloarcula amylolytica*, **b***H. japonica*, **c***H. hispanica* and **d***Haloarcula* sp. CBA1115. Yellow colour represents different positions between secondary structures of 16S rRNAs transcribed from *rrsA* and *rrsB* or *rrsC*. Gray colour represents gap between the sequences of 16S rRNA in *Haloarcula* strains and consensus sequence of archaeal 16S rRNA

**Fig. 2:**
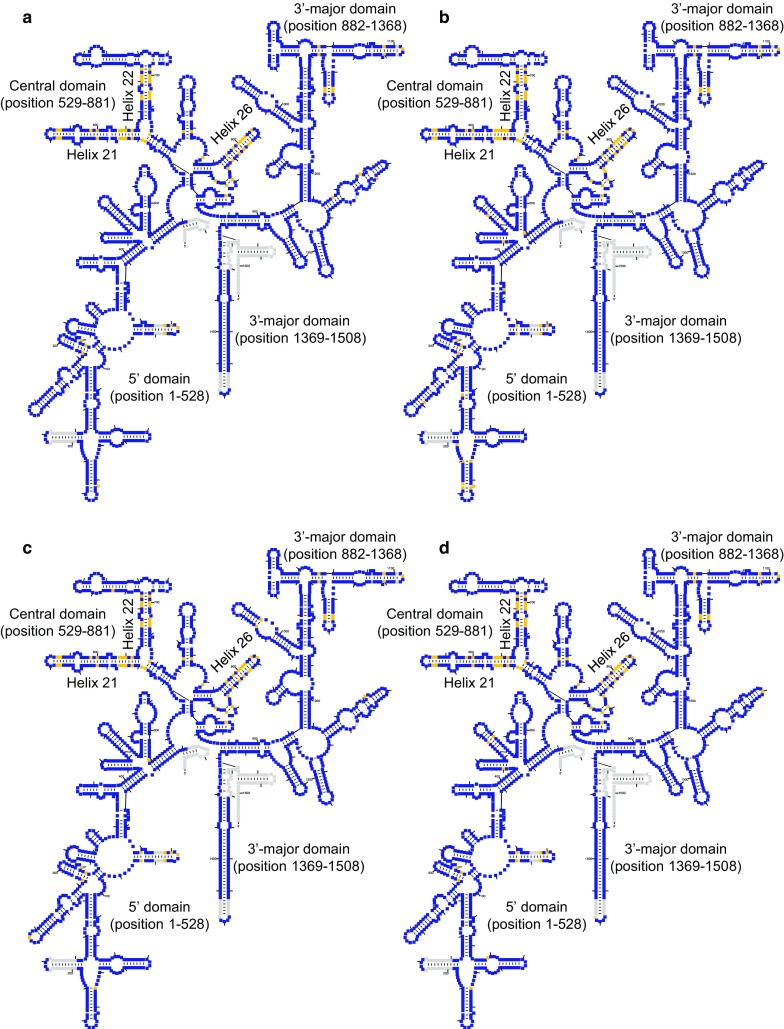
Secondary structure of 16S rRNAs in **a***Haloarcula argentinensis*, **b***H. quadrata*, **c***H. vallismortis* and **d** “*H. californiae*”. Yellow colour represents different positions between secondary structures of 16S rRNAs transcribed from *rrsA* and *rrsB*. Gray colour represents gap between the sequences of 16S rRNAs in *Haloarcula* strains and consensus sequence of archaeal 16S rRNA

We summarised different positions between secondary structures of 16S rRNAs predicted from *rrsA* and *rrsB* or *rrsC* in *Haloarcula* strains (Figs. 1, 2). The majority of differences (49–60%) were observed in helices 21, 22 and 26 of the central domain. A previous study showed that the affinity of ribosomal protein S8 to helix 21 in thermophiles and hyperthermophiles is much higher than that in mesophiles (Gruber et al. 2003). Furthermore, another study suggests that formation of helix 26 is essential for assembly of functional small subunit of ribosomes in *Escherichia coli* (Xu and Culver 2010). Therefore, these regions may be important in determining the functional differences among 16S rRNAs transcribed from *rrsA* and *rrsBC*.

The thermal stability of each 16S rRNA secondary structure was predicted with the use of the mfold web server (Table 3). The calculation results showed that the MFE values of the 16S rRNAs transcribed from *rrsA* were significantly lower than those of the 16S rRNAs transcribed from *rrsB* and *rrsC* under the same temperature conditions (*p *< 0.05 by Student’s *t* test). These results suggest that the thermal stability of the secondary structure of the 16S rRNAs transcribed from high-*P*_*GC*_*rrsA* is greater than those of the 16S rRNAs transcribed from low-*P*_*GC*_*rrsB* or *rrsC*.

**Table 3:** Minimum folding free energy values calculated from 16S rRNA sequences of *Haloarcula* strains at the minimum (*T*_*min*_), optimal (*T*_*opt*_) and maximum (*T*_*max*_) growth temperature

Strain	Type	Minimum folding free energy (kcal mol^−1^)		
		*T*_*min*_	*T*_*opt*_	*T*_*max*_
*Haloarcula* sp. CBA1115	*rrsA*	− 786.5	− 403.8	− 343.0
	*rrsB*	− 767.1	− 389.2	− 329.2
	*rrsC*	− 764.3	− 387.6	− 327.8
*Haloarcula argentinensis*	*rrsA*	− 784.1	− 461.9	− 401.7
	*rrsB*	− 762.9	− 442.2	− 381.2
*Haloarcula quadrata*	*rrsA*	− 768.3	− 512.0	− 450.9
	*rrsB*	− 759.2	− 503.0	− 441.1
*Haloarcula vallismortis*	*rrsA*	− 786.6	− 464.8	− 404.2
	*rrsB*	− 774.7	− 452.7	− 392.6
*Haloarcula amylolytica*	*rrsA*	− 776.2	− 460.2	− 400.3
	*rrsB*	− 766.0	− 448.0	− 388.7
	*rrsC*	− 756.7	− 440.7	− 381.8
*Haloarcula japonica*	*rrsA*	− 783.2	− 462.3	− 402.0
	*rrsC*	− 755.8	− 439.7	− 380.5
*Haloarcula hispanica*	*rrsA*	− 788.2	− 466.4	− 405.9
	*rrsB*	− 767.2	− 446.7	− 387.5
	*rrsC*	− 763.5	− 440.3	− 381.2
“*Haloarcula californiae*”	*rrsA*	− 780.1	− 523.0	− 461.4

### Expression of *rrsA* and *rrsBC* under various temperature conditions

The selectivity of the primer sets used in this study was verified by qPCR using PCR product of *rrsA*, *rrsB* or *rrsC* amplified from the clone libraries constructed in this study (Fig. S3). Proper products were amplified from only *rrsA* in *H. quadrata*, *rrsA* in *H. vallismortis* and *rrsA* in the other *Haloarcula* strains using the primer sets, rrsAf2/rrsAr, rrsAf/rrsAr2 and rrsAf/rrsAr, respectively. Proper products were amplified from only *rrsB* and *rrsC* in all *Haloarcula* strains using the primer set rrsBCf/rrsBCr. In addition, melting curve analyses showed that the melting temperature of the products from *rrsA* is about 1 °C higher than those of products from *rrsB* or *rrsC* in all *Haloarcula* strains. These results suggest that all of the primer sets were well designed to precisely amplify *rrsA* or *rrsBC*.

**Fig. 3:**
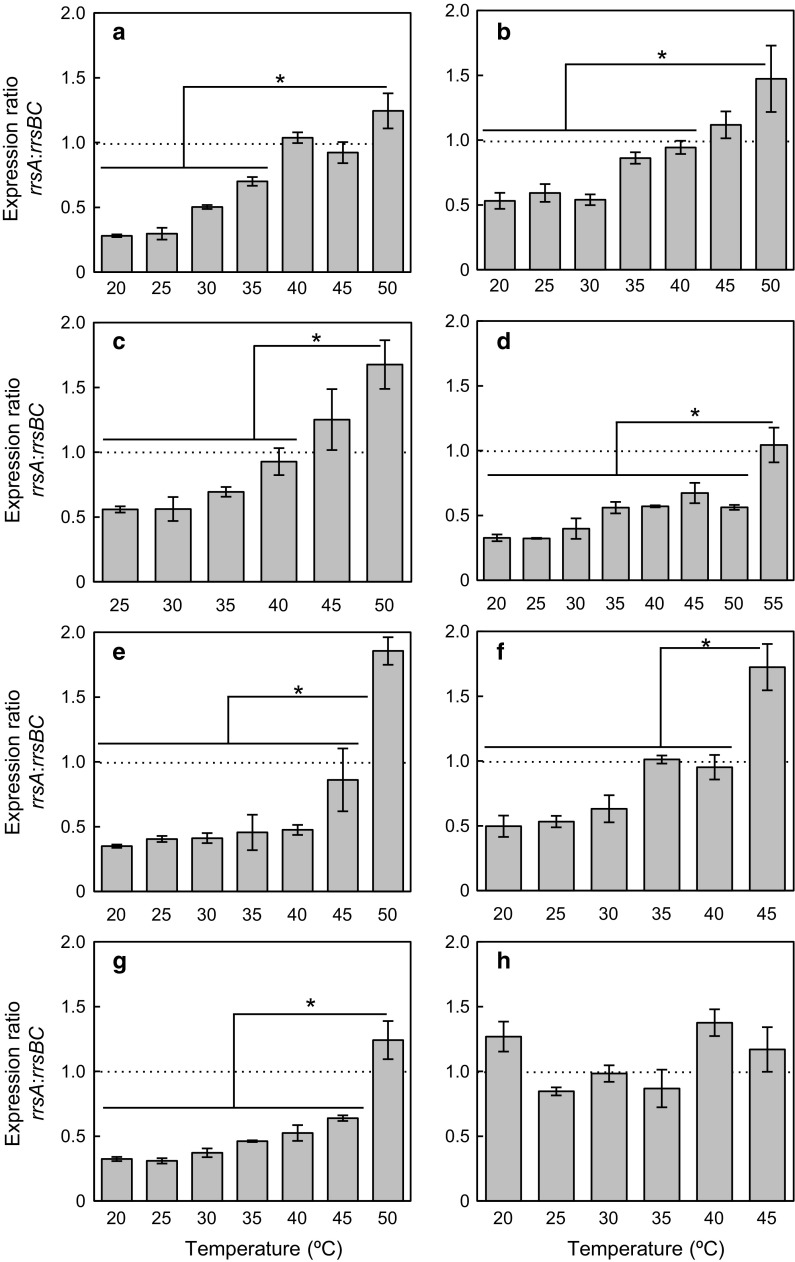
Expression ratio of *rrsA* to *rrsBC* (*rrsA*:*rrsBC*) under various temperature conditions in **a***Haloarcula amylolytica*, **b***H. japonica*, **c***H. hispanica*, **d***Haloarcula* sp. CBA1115, **e***H. argentinensis*, **f***H. quadrata*, **g***H. vallismortis* and **h** “*H. californiae*”. Error bars denote the standard deviation for triplicate measurements. The results for *H*. *hispanica* (**c**) are taken from Sato et al. (2017). Asterisks indicate significant differences among samples (Student’s *t* test, *p* < 0.05)

We quantified each 16S rRNA in *Haloarcula* strains by using RT-qPCR. No significant differences were found between biological replicates. The results of our RT-qPCR suggest that the expression levels of *rrsA* and *rrsBC* in seven of the *Haloarcula* strains varied depending on the incubation temperatures (Fig. 3). The expression ratio of *rrsA* to *rrsBC* (*rrsA*:*rrsBC* ratio) in particular increased with the cultivation temperature, and the maximum expression ratios were observed at each *T*_*max*_. We were able to divide the expression patterns in the *Haloarcula* strains into three types. First, the expression ratios continuously increased with temperature. This expression pattern was observed in *H. amylolytica*, *H. japonica* and *H. hispanica* (Fig. 3a–c). Although the expression ratio did not vary at temperatures close to *T*_*opt*_ and *T*_*max*_, the ratio at *T*_*max*_ was significantly higher than those below 40 °C (*p *< 0.05 by Student’s *t* test). Second, the expression ratios increased suddenly at *T*_*max*_ in *Haloarcula* sp. CBA1115, *H. argentinensis*, *H. quadrata* and *H. vallismortis* (Fig. 3d–g). This ratio at *T*_*max*_ was significantly higher than those at other tested temperatures (*p *< 0.05 by Student’s *t* test). Third, the expression ratio stayed constant under all tested temperatures in “*H. californiae*” (Fig. 3h).

Although we observed differences in the expression levels of *rrsA* and *rrsBC*, all eight of the *Haloarcula* strains simultaneously expressed these 16S rRNA genes at all tested temperatures. The *rrsA*:*rrsBC* ratio increased with temperature in seven of the eight *Haloarcula* strains (Fig. 3). A previous study using *H. marismortui* also showed that the expression ratio of a high-*P*_*GC*_ 16S rRNA gene compared to low-*P*_*GC*_ 16S rRNA genes increased markedly at *T*_*max*_ (50 °C) (López-López et al. 2007). Our present findings extend those of the previous study and suggest that the expression ratios of high-*P*_*GC*_ 16S rRNA genes to low-*P*_*GC*_ 16S rRNA genes increase with temperature in most *Haloarcula* strains. The variation in expression ratios may occur due to temperature-dependent expression of 16S rRNA genes and/or thermal degradation of 16S rRNAs with low stability transcribed from *rrsBC* at high temperatures. Moreover, we observed that the expression ratios at 20 °C and 25 °C ranged from 0.28 to 0.59 in seven *Haloarcula* strains, which suggests that the total expression of *rrsB* and *rrsC* was 1.7 to 3.6 times as much as that of *rrsA*. Low-*P*_*GC*_ 16S rRNA gene can be transcribed and replicated with low energy costs because of two reasons. First, costs for de novo production of adenine, thymine and uracil is lower than those of guanine and cytosine (Chen et al. 2016). Second, force for unwinding adenine–thymine rich region is lower than that for unwinding guanine-cytosine rich regions (Bockelmann et al. 1997; Essevaz-Roulet et al. 1997; Bockelmann et al. 1998; Rief et al. 1999). Therefore, low-*P*_*GC*_*rrsB* and *rrsC* in *Haloarcula* strains can help to reduce energy consumption in transcription and replication under low temperature conditions, which might explain why total expression of *rrsB* and *rrsC* increased at low temperatures close to the actual *T*_*min*_ in seven of the eight *Haloarcula* strains. Although the expression mechanisms of these 16S rRNA genes are unclear, they can be verified in future studies.

### High temperature adaptation of *Haloarcula* strains in their habitats

Sato et al. (2017) constructed rRNA operon double-mutant strains of *H. hispanica* that harbour only *rrnA* including high-*P*_*GC*_*rrsA* (58.9%) or only *rrnC* including low-*P*_*GC*_*rrsC* (56.4%). Both mutant strains grew slower than the wild-type strain at low temperature close to the *T*_*min*_, whereas the mutant harbouring *rrnA* containing only high-*P*_*GC*_*rrsA* grew as fast as the wild-type strain at high temperature close to the *T*_*opt*_ and *T*_*max*_. Their findings suggest that rRNA transcribed from the rRNA operon containing high-*P*_*GC*_*rrsA* is important for growth at high temperature in *H. hispanica*.

In the present study, the *rrsA*:*rrsBC* ratios increased with the incubation temperature in six *Haloarcula* strains as well as *H. hispanica* (Fig. 3). The results of the secondary structure prediction in silico indicate that 16S rRNAs transcribed from high-*P*_*GC*_*rrsA* in *Haloarcula* strains can form a more stable secondary structure at high and low temperatures compared to those transcribed from low-*P*_*GC*_*rrsBC*. In addition, only *Haloarcula* sp. CBA1115 harbouring the highest *P*_*GC*_ (59.0%) of 16S rRNA gene grew at 55 °C, the highest growth temperature among the *Haloarcula* strains reported previously (Bowers and Wiegel 2011; Namwong et al. 2011). These findings imply that 16S rRNAs transcribed from high-*P*_*GC*_*rrsA* are potentially important for growth at high temperatures close to actual *T*_*max*_ (45–55 °C) in the *Haloarcula* strains inhabiting solar salterns and salt lakes, where environmental temperature varies greatly in a day (Wieland et al. 2005; López-López et al. 2007; Sima et al. 2013; Andrade et al. 2015).

## Conclusion

In this study, we performed the sequencing of 16S rRNA genes in eight *Haloarcula* strains, and we estimated the growth temperatures using MMT based on the *P*_*GC*_ values of the 16S rRNA gene sequences. The estimated growth temperatures of cells carrying the high-*P*_*GC*_ 16S rRNA gene (*rrsA*) were approximately 6–15 °C higher than those carrying the low-*P*_GC_ 16S rRNA genes (*rrsB* and *rrsC*). In addition, the prediction analysis via computer simulation revealed that the stability of the secondary structure of 16S rRNAs transcribed from high-*P*_*GC*_*rrsA* was higher than that of 16S rRNAs transcribed from low-*P*_*GC*_*rrsBC* in the *Haloarcula* strains examined in this study, suggesting that small ribosomal subunits containing those 16S rRNAs may function effectively under various temperature conditions. These results suggest that *Haloarcula* strains harbour different sequences of 16S rRNA genes to maintain rapid growth over a wide range of temperatures.

We characterised the expression of *rrsA* and *rrsBC* of *H. hispanica* at different growth temperatures. We found that the expression ratio of high-*P*_*GC*_*rrsA* to low-*P*_*GC*_*rrsBC* increased with the incubation temperature in seven of the eight *Haloarcula* strains. Our results indicate that the temperature-dependent expression of 16S rRNA genes is a common feature in most *Haloarcula* strains. The environmental temperature in salt lakes and solar salterns varies in a day, and *Haloarcula* strains may express and utilise different *P*_*GC*_ of 16S rRNAs in response to variable temperature in their habitats.

## Electronic supplementary material

Below is the link to the electronic supplementary material.

Supplementary material 1 (DOC 991 kb)
