# Supplementary material for: Temperature-dependent expression of different guanine-plus-cytosine content 16S rRNA genes in Haloarcula strains of the class Halobacteria
Source: Antonie Van Leeuwenhoek. 2018 Aug 20;112(2):187–201. doi: 10.1007/s10482-018-1144-3 (PMC6373231; doi:10.1007/s10482-018-1144-3)
Supplement: Supplementary file 1 — Supplementary material 1 (DOC 991 kb) [file 10482_2018_1144_MOESM1_ESM.doc]

Electronic Supplementary Material

**Temperature-dependent expression of different guanine-plus-cytosine content 16S rRNA genes in *Haloarcula* strains of the class *Halobacteria***

**Yu Sato^1^ and Hiroyuki Kimura^1,2,3*^**

^1^Department of Environment and Energy Systems, Graduate School of Science and Technology, Shizuoka University, Shizuoka, Japan

^2^Department of Geosciences, Faculty of Science, Shizuoka University, Shizuoka, Japan

^3^Research Institute of Green Science and Technology, Shizuoka University, Shizuoka, Japan

*Correspondence

Hiroyuki Kimura

Department of Geosciences, Faculty of Science, Shizuoka University, Shizuoka, Japan

836 Oya, Suruga-ku, Shizuoka 422-8529, Japan

E-mail: kimura.hiroyuki@shizuoka.ac.jp

Tel: +81-54-238-4784

Fax: +81-54-238-0491

ORCID: 0000-0002-9358-2673

**Table S1** Sequence information of 16S rRNA genes in *Haloarcula* strains

| Strain | Type^a^ | Accession no. | BLAST result | |
| --- | --- | --- | --- | --- |
|  |  |  | Best hit | Identity (%) |
| *Haloarcula amylolytica* | *rrsA* | LC198784 | AB605776 | 100 |
|  | *rrsB* | LC198785 | DQ826513 | 99.7 |
|  | *rrsC* | LC198786 | DQ854818 | 99.9 |
| *Haloarcula japonica* | *rrsA* | LC085248 | AB355986 | 100 |
|  | *rrsC* | LC085249 | EF645686 | 100 |
| *Haloarcula hispanica* | *rrsA* | LC085245^b^ | CP002921 | 100 |
|  | *rrsB* | LC085246^b^ | CP002921 | 100 |
|  | *rrsC* | LC085247^b^ | CP002922 | 99.9 |
| *Haloarcula* sp. CBA1115 | *rrsA* | LC198781 | CP010529 | 100 |
|  | *rrsB* | LC198782 | CP010529 | 100 |
|  | *rrsC* | LC198783 | CP010529 | 100 |
| *Haloarcula argentinensis* | *rrsA* | LC198789 | AB355983 | 100 |
|  | *rrsB* | LC198790 | D50849 | 99.8 |
| *Haloarcula quadrata* | *rrsA* | LC198787 | AB605777 | 100 |
|  | *rrsB* | LC198788 | AB010964 | 99.2 |
| *Haloarcula vallismortis* | *rrsA* | LC198793 | AB355982 | 100 |
|  | *rrsB* | LC198794 | EF645688 | 99.9 |
| "*Haloarcula californiae*" | *rrsA* | LC198791 | AB605778 | 100 |
|  | *rrsB* | LC198792 | AB477984 | 99.5 |

^a^We defined *rrsA* as the highest-*P_GC_* one among the copies of 16S rRNA genes in each *Haloarcula* strain in this study.

^b^The sequences were obtained from Sato et al. (2017).

**Table S2** Sequence information in halophilic archaea with high intragenomic heterogeneity (1.0% and more) of 16S rRNA genes

| Strain | 16S rRNA gene | | | | Reference |
| --- | --- | --- | --- | --- | --- |
|  | Accession no. | Sequence | *P_GC_* | *P_GC_* |  |
|  |  | difference^a^ |  | offset^b^ |  |
|  |  | (%) | (%) | (%) |  |
| *Halorussus ruber* | JQ237116 | 4.4 | 59.6 | 1.7 | Xu et al. (2015) |
|  | KJ913074 |  | 57.9 |  |  |
| *Halomicroarcula limicola* | KF582944 | 4.1 | 59.6 | 1.7 | Zhang and Cui (2014a) |
|  | JQ237128 |  | 57.9 |  |  |
| *Halomicroarcula pellucida* | AB766179 | 5.1 | 58.6 | 1.6 | Echigo et al. (2013) |
|  | AB766180 |  | 57.0 |  |  |
| *Halomicrobium zhouii* | HQ215546 | 7.6 | 58.7 | 1.5 | Yang and Cui (2012) |
|  | HM063952 |  | 58.6 |  |  |
|  | HQ215547 |  | 57.2 |  |  |
| *Haloarchaeobius salinus* | JQ937361 | 4.7 | 58.4 | 1.5 | Yuan et al. (2015a) |
|  | KM386640 |  | 56.9 |  |  |
| *Natronoarchaeum rubrum* | GU951432 | 4.8 | 61.4 | 1.4 | Qiu et al. (2014) |
|  | JF421970 |  | 60.0 |  |  |
| *Halomicroarcula salina* | KF582943 | 3.0 | 58.4 | 1.4 | Zhang and Cui (2015) |
|  | JQ237127 |  | 57.0 |  |  |
| *Haloarchaeobius litoreus* | GU951427 | 4.7 | 58.3 | 1.1 | Zhang and Cui (2014b) |
|  | JF421971 |  | 57.2 |  |  |
| *Halomicrobium katesii* | JN120801 | 7.3 | 58.9 | 1.0 | Kharroub et al. (2008) |
|  | JN120802 |  | 58.0 |  |  |
|  | JN120803 |  | 57.9 |  |  |
| *Halosimplex rubrum* | KF434757 | 6.8 | 59.5 | 1.0 | Han and Cui (2014) |
|  | HM159603 |  | 58.5 |  |  |
| *Natrinema* sp. XA3-1 | AJ586110 | 5.0 | 58.9 | 1.0 | Boucher et al. (2004) |
|  | AJ586111 |  | 58.9 |  |  |
|  | AJ586112 |  | 58.9 |  |  |
|  | AJ586109 |  | 57.9 |  |  |
| *Haladaptatus paucihalophilus* | DQ344973 | 4.3 | 57.5 | 1.0 | Savage et al. (2007) |
|  | DQ344974 |  | 56.5 |  |  |
| *Halomicrobium mukohataei* | EF645690 | 8.7 | 59.1 | 0.9 | Cui et al. (2009) |
|  | EF645691 |  | 58.2 |  |  |
| *Halorussus amylolyticus* | JQ237117 | 2.3 | 58.9 | 0.9 | Yuan et al. (2015b) |
|  | KM368828 |  | 58.0 |  |  |
| **Table S2** *Continued* |  |  |  |  |  |
| Strain | 16S rRNA gene | | | | Reference |
|  | Accession no. | Sequence | *P_GC_* | *P_GC_* |  |
|  |  | difference^a^ |  | offset^b^ |  |
|  |  | (%) | (%) | (%) |  |
| *Halosimplex pelagicum* | KF434756 | 4.5 | 59.5 | 0.8 | Han and Cui (2014) |
|  | HM159602 |  | 58.7 |  |  |
| *Halobaculum magnesiiphilum* | AB627357 | 2.4 | 59.1 | 0.7 | Shimoshige et al. (2013) |
|  | AB638780 |  | 58.4 |  |  |
| *Halorubrum halophilum* | EF077637 | 1.1 | 59.3 | 0.7 | Yim et al. (2014) |
|  | KF848218 |  | 59.1 |  |  |
|  | KF848217 |  | 58.6 |  |  |
| *Halosimplex litoreum* | KM386641 | 3.9 | 60.1 | 0.6 | Yuan et al. (2015c) |
|  | JQ937362 |  | 59.5 |  |  |
| *Halosimplex carlsbadense*^c^ | AF320480 | 5.8 | 59.3 | 0.6 | Vreeland et al. (2002) |
|  | AF320479 |  | 58.7 |  |  |
| *Haloarchaeobius baliensis* | LC061269 | 5.3 | 58.2 | 0.4 | Mori et al. (2016) |
|  | LC061270 |  | 57.8 |  |  |
| *Halobaculum roseum* | KX376700 | 3.9 | 59.0 | 0.4 | Chen et al. (2017) |
|  | KX376701 |  | 58.6 |  |  |
| *Haloarchaeobius iranensis* | JF293278 | 1.6 | 58.3 | 0.4 | Makhdoumi-Kakhki et al. (2012) |
|  | JF293279 |  | 57.9 |  |  |
| *Halorubellus salinus* | GU951429 | 3.9 | 58.1 | 0.2 | Cui et al. (2012) |
|  | HQ236376 |  | 57.9 |  |  |
| *Haloparvum sedimenti* | KP202830 | 1.0 | 59.4 | 0.2 | Chen et al. (2016) |
|  | KP202831 |  | 59.2 |  |  |
| *Halorubellus litoreus* | HQ236377 | 2.4 | 58.2 | 0.1 | Cui et al. (2012) |
|  | GU951430 |  | 58.1 |  |  |

^a^Maximum sequence difference among 16S rRNA genes in each strain.

^b^Maximum *P_GC_* offset among 16S rRNA genes in each strain.

^c^One of 16S rRNA genes (AF320478) in *Halosimplex carlsbadense* was removed because Boucher et al. (2004) demonstrated that it was PCR chimeric products.

**Fig. S1** Growth rates at each temperature in in (a) *Haloarcula amylolytica*, (b) *H. japonica*, (c) *H. hispanica*, (d) *Haloarcula* sp. CBA1115, (e) *H. argentinensis*, (f) *H. quadrata*, (g) *H. vallismortis* and (h) “*H. californiae*”.

**Fig. S2** Maximum OD_660_ at each temperature in in (a) *Haloarcula amylolytica*, (b) *H. japonica*, (c) *H. hispanica*, (d) *Haloarcula* sp. CBA1115, (e) *H. argentinensis*, (f) *H. quadrata*, (g) *H. vallismortis* and (h) “*H. californiae*”.

**Fig. S3** Electrophoresis results of PCR products after qPCR. qPCR was performed by using 100-fold diluted PCR products (109aF/915aR) of *rrsA*, *rrsB* and *rrsC* in *Haloarcula* strains. The number of PCR cycles are fourteen. Proper products (127 bp) were amplified from only *rrsA* in *H. quadrata*, *rrsA* in *H. vallismortis* and *rrsA* in the other *Haloarcula* strains using the primer sets, rrsAf2/rrsAr, rrsAf/rrsAr2 and rrsAf/rrsAr, respectively. Proper products (132 bp) were amplified from only *rrsB* and *rrsC* in all *Haloarcula* strains using the primer set rrsBCf/rrsBCr. Abbreviations: Amy, *Haloarcula amylolytica*; Jpn, *H. japonica*; His, *H. hispanica*; CBA, *Haloarcula* sp. strain CBA1115; Arg, *H. argentinensis*; Qua, *H. quadrata*; Val, *H. vallismortis*; Cal, "*H. californiae*"; A, B and C; qPCR results with PCR products of *rrsA*, *rrsB* and *rrsC*, respectively; N, negative control; M, marker, 20 bp DNA ladder (Takara Bio, Shiga, Japan).

**Supplementary References**

Boucher Y, Douady CJ, Sharma AK, Kamekura M, Doolittle WF (2004) Intragenomic heterogeneity and intergenomic recombination among haloarchaeal rRNA genes. J Bacteriol 186:3980–3990. doi: 10.1128/JB.186.12.3980-3990.2004

Chen S, Liu HC, Zhou J, Xiang H (2016) *Haloparvum sedimenti* gen. nov., sp. nov., a member of the family *Haloferacaceae*. Int J Syst Evol Microbiol 66:2327–2334. doi: 10.1099/ijsem.0.001033

Chen S, Xu Y, Liu H-C, Yang A-N, Ke L-X (2017) *Halobaculum roseum* sp. nov., isolated from underground salt deposits. Int J Syst Evol Microbiol 67:818–823. doi: 10.1099/ijsem.0.001663

Cui HL, Mou YZ, Yang X, Zhou YG, Liu HC, Zhou PJ (2012) *Halorubellus salinus* gen. nov., sp. nov. and *Halorubellus litoreus* sp. nov., novel halophilic archaea isolated from a marine solar saltern. Syst Appl Microbiol 35:30–34. doi: 10.1016/j.syapm.2011.08.001

Cui HL, Zhou PJ, Oren A, Liu SJ (2009) Intraspecific polymorphism of 16S rRNA genes in two halophilic archaeal genera, *Haloarcula* and *Halomicrobium*. Extremophiles 13:31–37. doi: 10.1007/s00792-008-0194-2

Echigo A, Minegishi H, Shimane Y, Kamekura M, Itoh T, Usami R (2013) *Halomicroarcula pellucida* gen. nov., sp. nov., a non-pigmented, transparent-colony-forming, halophilic archaeon isolated from solar salt. Int J Syst Evol Microbiol 63:3556–3562. doi: 10.1099/ijs.0.049965-0

Han D, Cui H-L (2014) *Halosimplex pelagicum* sp. nov. and *Halosimplex rubrum* sp. nov., isolated from salted brown alga *Laminaria*, and emended description of the genus *Halosimplex*. Int J Syst Evol Microbiol 64:169–173. doi: 10.1099/ijs.0.056887-0

Kharroub K, Lizama C, Aguilera M, Boulahrouf A, Campos V, Ramos-Cormenzana A, Monteoliva-Sánchez M (2008) *Halomicrobium katesii* sp. nov., an extremely halophilic archaeon. Int J Syst Evol Microbiol 58:2354–2358. doi: 10.1099/ijs.0.65662-0

Makhdoumi-Kakhki A, Amoozegar MA, Bagheri M, Ramezani M, Ventosa A (2012) *Haloarchaeobius iranensis* gen. nov., sp. nov., an extremely halophilic archaeon isolated from a saline lake. Int J Syst Evol Microbiol 62:1021–1026. doi: 10.1099/ijs.0.033167-0

Mori K, Suzuki K, Yopi H, Nurcahyanto DA, Lisdiyanti P, Kawasaki H (2016) *Haloarchaeobius baliensis* sp. nov., isolated from a solar saltern. Int J Syst Evol Microbiol 66:38–43. doi: 10.1099/ijsem.0.000672

Qiu XX, Zhao ML, Cui HL (2014) *Natronoarchaeum rubrum* sp. nov., isolated from a marine solar saltern, and emended description of the genus *Natronoarchaeum*. Int J Syst Evol Microbiol 64:952–956. doi: 10.1099/ijs.0.059071-0

Sato Y, Fujiwara T, Kimura H (2017) Expression and function of different guanine-plus-cytosine content 16S rRNA genes in *Haloarcula hispanica* at different temperatures. Front Microbiol 8:482. doi: 10.3389/fmicb.2017.00482

Savage KN, Krumholz LR, Oren A, Elsahed MS (2007) *Haladaptatus paucihalophilus* gen. nov., sp. nov., a halophilic archaeon isolated from a low-salt, sulfide-rich spring. Int J Syst Evol Microbiol 57:19–24. doi: 10.1099/ijs.0.64464-0

Shimoshige H, Yamada T, Minegishi H, Echigo A, Shimane Y, Kamekura M, Itoh T, Usami R (2013) *Halobaculum magnesiiphilum* sp. nov., a magnesium-dependent haloarchaeon isolated from commercial salt. Int J Syst Evol Microbiol 63:861–866. doi: 10.1099/ijs.0.037432-0

Vreeland RH, Straight S, Krammes J, Dougherty K, Rosenzweig WD, Kamekura M (2002) *Halosimplex carlsbadense* gen. nov., sp. nov., a unique halophilic archaeon, with three 16S rRNA genes, that grows only in defined medium with glycerol and acetate or pyruvate. Extremophiles 6:445–452. doi: 10.1007/s00792-002-0278-3

Xu WD, Zhang WJ, Han D, Cui HL, Yang K (2014) *Halorussus ruber* sp. nov. isolated from an inland salt lake of China. Arch Microbiol 197:91–95. doi: 10.1007/s00203-014-1058-z

Yang X, Cui HL (2012) *Halomicrobium zhouii* sp. nov., a halophilic archaeon from a marine solar saltern. Int J Syst Evol Microbiol 62:1235–1240. doi: 10.1099/ijs.0.031989-0

Yim KJ, Cha IT, Lee HW, Song HS, Kim KN, Lee SJ, Nam Y Do, Hyun DW, Bae JW, Rhee SK, Seo MJ, Choi JS, Choi HJ, Roh SW, Kim D (2014) *Halorubrum halophilum* sp. nov., an extremely halophilic archaeon isolated from a salt-fermented seafood. Antonie van Leeuwenhoek 105:603–612. doi: 10.1007/s10482-014-0115-6

Yuan PP, Xu JQ, Xu WM, Wang Z, Yin S, Han D, Zhang WJ, Cui HL (2015c) *Halosimplex litoreum* sp. nov., isolated from a marine solar saltern. Antonie van Leeuwenhoek 108:483–489. doi: 10.1007/s10482-015-0501-8

Yuan PP, Ye WT, Pan JX, Han D, Zhang WJ, Cui HL (2015b) *Halorussus amylolyticus* sp. nov., isolated from an inland salt lake. Int J Syst Evol Microbiol 65:3734–3738. doi: 10.1099/ijsem.0.000487

Yuan PP, Zhang WJ, Han D, Cui HL (2015a) *Haloarchaeobius salinus* sp. nov., isolated from an inland salt lake, and emended description of the genus *Haloarchaeobius*. Int J Syst Evol Microbiol 65:910–914. doi: 10.1099/ijs.0.000038

Zhang WJ, Cui HL (2014b) *Haloarchaeobius litoreus* sp. nov., isolated from a marine solar saltern. Antonie van Leeuwenhoek 105:1085–1090. doi: 10.1007/s10482-014-0166-8

Zhang WJ, Cui HL (2014a) *Halomicroarcula limicola* sp. nov., isolated from a marine solar saltern, and emended description of the genus *Halomicroarcula*. Int J Syst Evol Microbiol 64:1747–1751. doi: 10.1099/ijs.0.062455-0

Zhang WJ, Cui HL (2015) *Halomicroarcula salina* sp. nov., isolated from a marine solar saltern. Int J Syst Evol Microbiol 65:1628–1633. doi: 10.1099/ijs.0.000150
